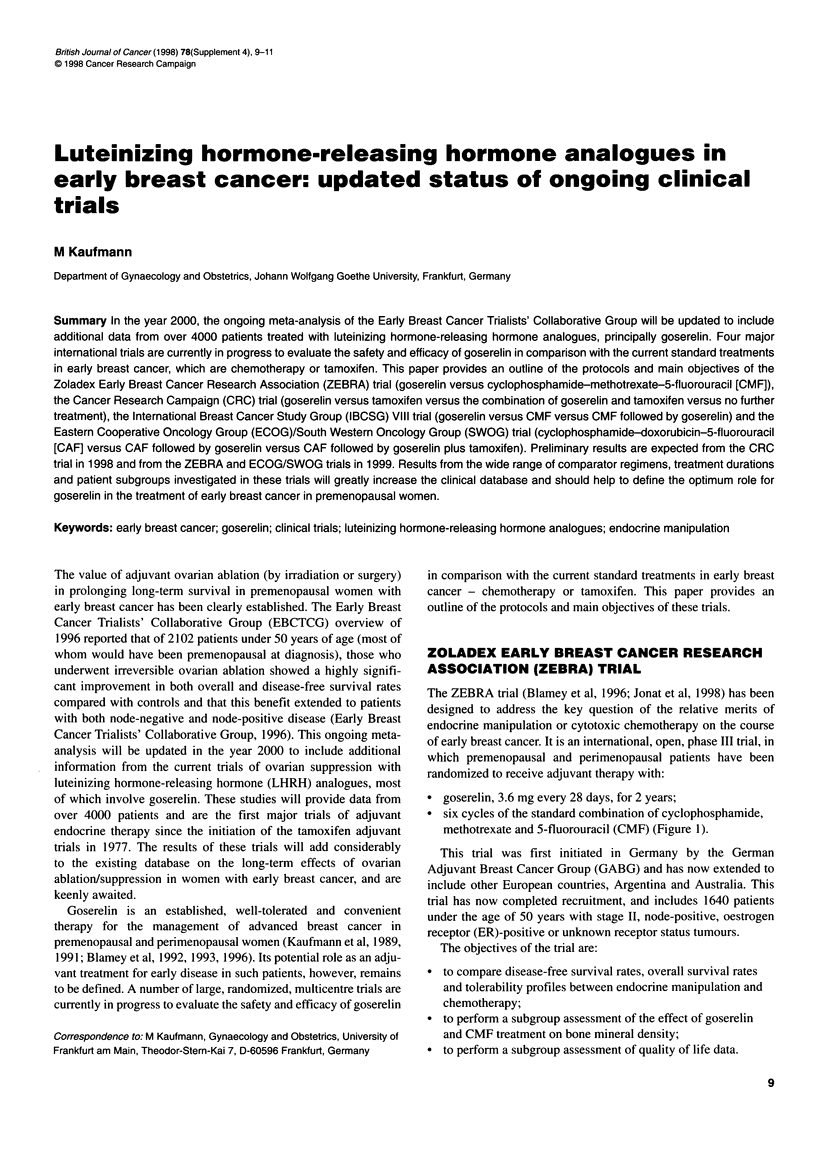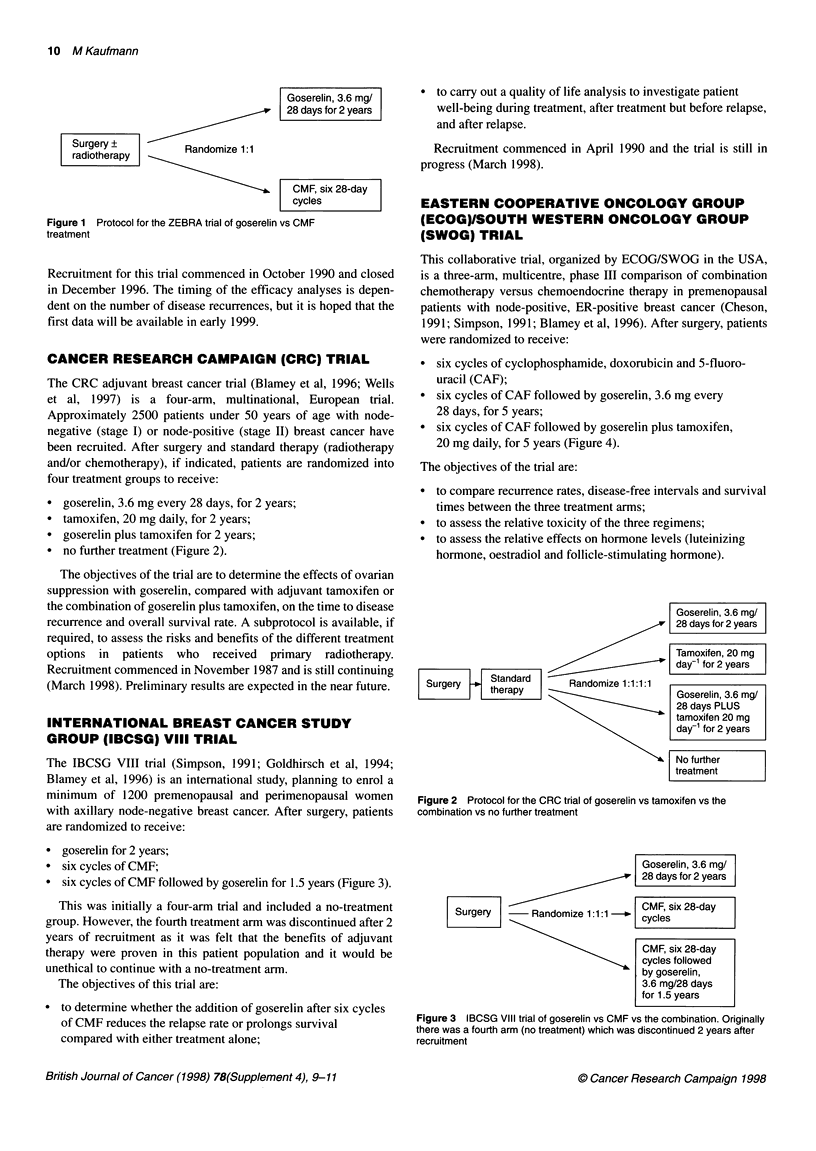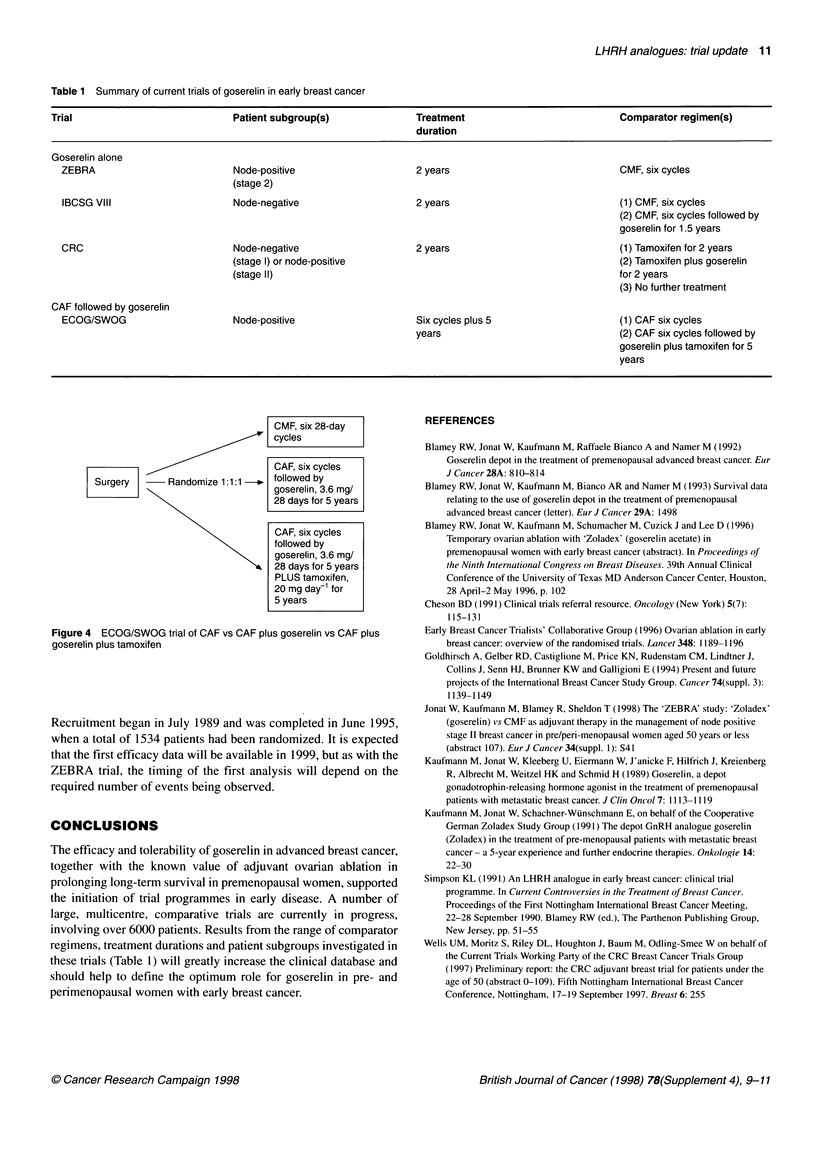# Luteinizing hormone-releasing hormone analogues in early breast cancer: updated status of ongoing clinical trials.

**DOI:** 10.1038/bjc.1998.755

**Published:** 1998-09

**Authors:** M. Kaufmann

**Affiliations:** Department of Gynaecology and Obstetrics, Johann Wolfgang Goethe University, Frankfurt, Germany.

## Abstract

In the year 2000, the ongoing meta-analysis of the Early Breast Cancer Trialists' Collaborative Group will be updated to include additional data from over 4000 patients treated with luteinizing hormone-releasing hormone analogues, principally goserelin. Four major international trials are currently in progress to evaluate the safety and efficacy of goserelin in comparison with the current standard treatments in early breast cancer, which are chemotherapy or tamoxifen. This paper provides an outline of the protocols and main objectives of the Zoladex Early Breast Cancer Research Association (ZEBRA) trial (goserelin versus cyclophosphamide-methotrexate-5-fluorouracil [CMF]), the Cancer Research Campaign (CRC) trial (goserelin versus tamoxifen versus the combination of goserelin and tamoxifen versus no further treatment), the International Breast Cancer Study Group (IBCSG) VIII trial (goserelin versus CMF versus CMF followed by goserelin) and the Eastern Cooperative Oncology Group (ECOG)/South Western Oncology Group (SWOG) trial (cyclophosphamide-doxorubicin-5-fluorouracil [CAF] versus CAF followed by goserelin versus CAF followed by goserelin plus tamoxifen). Preliminary results are expected from the CRC trial in 1998 and from the ZEBRA and ECOG/SWOG trials in 1999. Results from the wide range of comparator regimens, treatment durations and patient subgroups investigated in these trials will greatly increase the clinical database and should help to define the optimum role for goserelin in the treatment of early breast cancer in premenopausal women.


British Journal of Cancer (1998) 78(Supplement 4), 9–11
© 1998 Cancer Research Campaign

### Luteinizing hormone-releasing hormone analogues in early breast cancer: updated status of ongoing clinical trials

**M Kaufmann**

Department of Gynaecology and Obstetrics, Johann Wolfgang Goethe University, Frankfurt, Germany

**Summary** In the year 2000, the ongoing meta-analysis of the Early Breast Cancer Trialists' Collaborative Group will be updated to include additional data from over 4000 patients treated with luteinizing hormone-releasing hormone analogues, principally goserelin. Four major international trials are currently in progress to evaluate the safety and efficacy of goserelin in comparison with the current standard treatments in early breast cancer, which are chemotherapy or tamoxifen. This paper provides an outline of the protocols and main objectives of the Zoladex Early Breast Cancer Research Association (ZEBRA) trial (goserelin versus cyclophosphamide–methotrexate–5-fluorouracil [CMF]), the Cancer Research Campaign (CRC) trial (goserelin versus tamoxifen versus the combination of goserelin and tamoxifen versus no further treatment), the International Breast Cancer Study Group (IBCSG) VIII trial (goserelin versus CMF versus CMF followed by goserelin) and the Eastern Cooperative Oncology Group (ECOG)/South Western Oncology Group (SWOG) trial (cyclophosphamide–doxorubicin–5-fluorouracil [CAF] versus CAF followed by goserelin versus CAF followed by goserelin plus tamoxifen). Preliminary results are expected from the CRC trial in 1998 and from the ZEBRA and ECOG/SWOG trials in 1999. Results from the wide range of comparator regimens, treatment durations and patient subgroups investigated in these trials will greatly increase the clinical database and should help to define the optimum role for goserelin in the treatment of early breast cancer in premenopausal women.

**Keywords:** early breast cancer; goserelin; clinical trials; luteinizing hormone-releasing hormone analogues; endocrine manipulation

The value of adjuvant ovarian ablation (by irradiation or surgery) in prolonging long-term survival in premenopausal women with early breast cancer has been clearly established. The Early Breast Cancer Trialists' Collaborative Group (EBCTCG) overview of 1996 reported that of 2102 patients under 50 years of age (most of whom would have been premenopausal at diagnosis), those who underwent irreversible ovarian ablation showed a highly significant improvement in both overall and disease-free survival rates compared with controls and that this benefit extended to patients with both node-negative and node-positive disease (Early Breast Cancer Trialists' Collaborative Group, 1996). This ongoing meta-analysis will be updated in the year 2000 to include additional information from the current trials of ovarian suppression with luteinizing hormone-releasing hormone (LHRH) analogues, most of which involve goserelin. These studies will provide data from over 4000 patients and are the first major trials of adjuvant endocrine therapy since the initiation of the tamoxifen adjuvant trials in 1977. The results of these trials will add considerably to the existing database on the long-term effects of ovarian ablation/suppression in women with early breast cancer, and are keenly awaited.

Goserelin is an established, well-tolerated and convenient therapy for the management of advanced breast cancer in premenopausal and perimenopausal women (Kaufmann et al, 1989, 1991; Blamey et al, 1992, 1993, 1996). Its potential role as an adjuvant treatment for early disease in such patients, however, remains to be defined. A number of large, randomized, multicentre trials are currently in progress to evaluate the safety and efficacy of goserelin in comparison with the current standard treatments in early breast cancer – chemotherapy or tamoxifen. This paper provides an outline of the protocols and main objectives of these trials.

*Correspondence to:* M Kaufmann, Gynaecology and Obstetrics, University of Frankfurt am Main, Theodor-Stern-Kai 7, D-60596 Frankfurt, Germany

### ZOLADEX EARLY BREAST CANCER RESEARCH ASSOCIATION (ZEBRA) TRIAL

The ZEBRA trial (Blamey et al, 1996; Jonat et al, 1998) has been designed to address the key question of the relative merits of endocrine manipulation or cytotoxic chemotherapy on the course of early breast cancer. It is an international, open, phase III trial, in which premenopausal and perimenopausal patients have been randomized to receive adjuvant therapy with:

- goserelin, 3.6 mg every 28 days, for 2 years;
- six cycles of the standard combination of cyclophosphamide, methotrexate and 5-fluorouracil (CMF) (Figure 1).

This trial was first initiated in Germany by the German Adjuvant Breast Cancer Group (GABG) and has now extended to include other European countries, Argentina and Australia. This trial has now completed recruitment, and includes 1640 patients under the age of 50 years with stage II, node-positive, oestrogen receptor (ER)-positive or unknown receptor status tumours.

The objectives of the trial are:

- to compare disease-free survival rates, overall survival rates and tolerability profiles between endocrine manipulation and chemotherapy;
- to perform a subgroup assessment of the effect of goserelin and CMF treatment on bone mineral density;
- to perform a subgroup assessment of quality of life data.

**Figure 1** Protocol for the ZEBRA trial of goserelin vs CMF treatment

Recruitment for this trial commenced in October 1990 and closed in December 1996. The timing of the efficacy analyses is dependent on the number of disease recurrences, but it is hoped that the first data will be available in early 1999.

### CANCER RESEARCH CAMPAIGN (CRC) TRIAL

The CRC adjuvant breast cancer trial (Blamey et al, 1996; Wells et al, 1997) is a four-arm, multinational, European trial. Approximately 2500 patients under 50 years of age with node-negative (stage I) or node-positive (stage II) breast cancer have been recruited. After surgery and standard therapy (radiotherapy and/or chemotherapy), if indicated, patients are randomized into four treatment groups to receive:

- goserelin, 3.6 mg every 28 days, for 2 years;
- tamoxifen, 20 mg daily, for 2 years;
- goserelin plus tamoxifen for 2 years;
- no further treatment (Figure 2).

The objectives of the trial are to determine the effects of ovarian suppression with goserelin, compared with adjuvant tamoxifen or the combination of goserelin plus tamoxifen, on the time to disease recurrence and overall survival rate. A subprotocol is available, if required, to assess the risks and benefits of the different treatment options in patients who received primary radiotherapy. Recruitment commenced in November 1987 and is still continuing (March 1998). Preliminary results are expected in the near future.

### INTERNATIONAL BREAST CANCER STUDY GROUP (IBCSG) VIII TRIAL

The IBCSG VIII trial (Simpson, 1991; Goldhirsch et al, 1994; Blamey et al, 1996) is an international study, planning to enrol a minimum of 1200 premenopausal and perimenopausal women with axillary node-negative breast cancer. After surgery, patients are randomized to receive:

- goserelin for 2 years;
- six cycles of CMF;
- six cycles of CMF followed by goserelin for 1.5 years (Figure 3).

This was initially a four-arm trial and included a no-treatment group. However, the fourth treatment arm was discontinued after 2 years of recruitment as it was felt that the benefits of adjuvant therapy were proven in this patient population and it would be unethical to continue with a no-treatment arm.

The objectives of this trial are:

- to determine whether the addition of goserelin after six cycles of CMF reduces the relapse rate or prolongs survival compared with either treatment alone;
- to carry out a quality of life analysis to investigate patient well-being during treatment, after treatment but before relapse, and after relapse.

Recruitment commenced in April 1990 and the trial is still in progress (March 1998).

### EASTERN COOPERATIVE ONCOLOGY GROUP (ECOG)/SOUTH WESTERN ONCOLOGY GROUP (SWOG) TRIAL

This collaborative trial, organized by ECOG/SWOG in the USA, is a three-arm, multicentre, phase III comparison of combination chemotherapy versus chemoendocrine therapy in premenopausal patients with node-positive, ER-positive breast cancer (Cheson, 1991; Simpson, 1991; Blamey et al, 1996). After surgery, patients were randomized to receive:

- six cycles of cyclophosphamide, doxorubicin and 5-fluorouracil (CAF);
- six cycles of CAF followed by goserelin, 3.6 mg every 28 days, for 5 years;
- six cycles of CAF followed by goserelin plus tamoxifen, 20 mg daily, for 5 years (Figure 4).

The objectives of the trial are:

- to compare recurrence rates, disease-free intervals and survival times between the three treatment arms;
- to assess the relative toxicity of the three regimens;
- to assess the relative effects on hormone levels (luteinizing hormone, oestradiol and follicle-stimulating hormone).

**Figure 2** Protocol for the CRC trial of goserelin vs tamoxifen vs the combination vs no further treatment

**Figure 3** IBCSG VIII trial of goserelin vs CMF vs the combination. Originally there was a fourth arm (no treatment) which was discontinued 2 years after recruitment

© Cancer Research Campaign 1998

**Table 1** Summary of current trials of goserelin in early breast cancer

| Trial | Patient subgroup(s) | Treatment duration | Comparator regimen(s) |
|---|---|---|---|
| Goserelin alone | | | |
| ZEBRA | Node-positive (stage 2) | 2 years | CMF, six cycles |
| IBCSG VIII | Node-negative | 2 years | (1) CMF, six cycles<br>(2) CMF, six cycles followed by goserelin for 1.5 years |
| CRC | Node-negative (stage I) or node-positive (stage II) | 2 years | (1) Tamoxifen for 2 years<br>(2) Tamoxifen plus goserelin for 2 years<br>(3) No further treatment |
| CAF followed by goserelin | | | |
| ECOG/SWOG | Node-positive | Six cycles plus 5 years | (1) CAF six cycles<br>(2) CAF six cycles followed by goserelin plus tamoxifen for 5 years |

**Figure 4** ECOG/SWOG trial of CAF vs CAF plus goserelin vs CAF plus goserelin plus tamoxifen

Recruitment began in July 1989 and was completed in June 1995, when a total of 1534 patients had been randomized. It is expected that the first efficacy data will be available in 1999, but as with the ZEBRA trial, the timing of the first analysis will depend on the required number of events being observed.

### CONCLUSIONS

The efficacy and tolerability of goserelin in advanced breast cancer, together with the known value of adjuvant ovarian ablation in prolonging long-term survival in premenopausal women, supported the initiation of trial programmes in early disease. A number of large, multicentre, comparative trials are currently in progress, involving over 6000 patients. Results from the range of comparator regimens, treatment durations and patient subgroups investigated in these trials (Table 1) will greatly increase the clinical database and should help to define the optimum role for goserelin in pre- and perimenopausal women with early breast cancer.

### REFERENCES

Blamey RW, Jonat W, Kaufmann M, Raffaele Bianco A and Namer M (1992) Goserelin depot in the treatment of premenopausal advanced breast cancer. *Eur J Cancer* **28A**: 810–814

Blamey RW, Jonat W, Kaufmann M, Bianco AR and Namer M (1993) Survival data relating to the use of goserelin depot in the treatment of premenopausal advanced breast cancer (letter). *Eur J Cancer* **29A**: 1498

Blamey RW, Jonat W, Kaufmann M, Schumacher M, Cuzick J and Lee D (1996) Temporary ovarian ablation with 'Zoladex' (goserelin acetate) in premenopausal women with early breast cancer (abstract). In *Proceedings of the Ninth International Congress on Breast Diseases*. 39th Annual Clinical Conference of the University of Texas MD Anderson Cancer Center, Houston, 28 April–2 May 1996, p. 102

Cheson BD (1991) Clinical trials referral resource. *Oncology* (New York) **5**(7): 115–131

Early Breast Cancer Trialists' Collaborative Group (1996) Ovarian ablation in early breast cancer: overview of the randomised trials. *Lancet* **348**: 1189–1196

Goldhirsch A, Gelber RD, Castiglione M, Price KN, Rudenstam CM, Lindtner J, Collins J, Senn HJ, Brunner KW and Galligioni E (1994) Present and future projects of the International Breast Cancer Study Group. *Cancer* **74**(suppl. 3): 1139–1149

Jonat W, Kaufmann M, Blamey R, Sheldon T (1998) The 'ZEBRA' study: 'Zoladex' (goserelin) *vs* CMF as adjuvant therapy in the management of node positive stage II breast cancer in pre/peri-menopausal women aged 50 years or less (abstract 107). *Eur J Cancer* **34**(suppl. 1): S41

Kaufmann M, Jonat W, Kleeberg U, Eiermann W, J'anicke F, Hilfrich J, Kreienberg R, Albrecht M, Weitzel HK and Schmid H (1989) Goserelin, a depot gonadotrophin-releasing hormone agonist in the treatment of premenopausal patients with metastatic breast cancer. *J Clin Oncol* **7**: 1113–1119

Kaufmann M, Jonat W, Schachner-Wünschmann E, on behalf of the Cooperative German Zoladex Study Group (1991) The depot GnRH analogue goserelin (Zoladex) in the treatment of pre-menopausal patients with metastatic breast cancer – a 5-year experience and further endocrine therapies. *Onkologie* **14**: 22–30

Simpson KL (1991) An LHRH analogue in early breast cancer: clinical trial programme. In *Current Controversies in the Treatment of Breast Cancer*. Proceedings of the First Nottingham International Breast Cancer Meeting, 22–28 September 1990. Blamey RW (ed.), The Parthenon Publishing Group, New Jersey, pp. 51–55

Wells UM, Moritz S, Riley DL, Houghton J, Baum M, Odling-Smee W on behalf of the Current Trials Working Party of the CRC Breast Cancer Trials Group (1997) Preliminary report: the CRC adjuvant breast trial for patients under the age of 50 (abstract 0–109). Fifth Nottingham International Breast Cancer Conference, Nottingham, 17–19 September 1997. *Breast* **6**: 255

© *Cancer Research Campaign 1998*